# Beyond the looking glass: recent advances in understanding the impact of environmental exposures on neuropsychiatric disease

**DOI:** 10.1038/s41386-020-0648-5

**Published:** 2020-02-28

**Authors:** Jonathan A. Hollander, Deborah A. Cory-Slechta, Felice N. Jacka, Steven T. Szabo, Tomás R. Guilarte, Staci D. Bilbo, Carolyn J. Mattingly, Sheryl S. Moy, Ebrahim Haroon, Mady Hornig, Edward D. Levin, Mikhail V. Pletnikov, Julia L. Zehr, Kimberly A. McAllister, Anika L. Dzierlenga, Amanda E. Garton, Cindy P. Lawler, Christine Ladd-Acosta

**Affiliations:** 1 Genes, Environment and Health Branch, National Institute of Environmental Health Sciences, NIH, Research Triangle Park, NC USA; 2 Department of Environmental Medicine, Box EHSC, University of Rochester Medical Center, Rochester, NY USA; 3 Food & Mood Centre, IMPACT SRC, School of Medicine, Deakin University, Geelong, VIC Australia; 4 iMPACT (the Institute for Mental and Physical Health and Clinical Translation), Food & Mood Centre, Deakin University, Geelong, VIC Australia; 5 Centre for Adolescent Health, Murdoch Children’s Research Institute, Melbourne, VIC Australia; 6 Black Dog Institute, Sydney, NSW Australia; 7 James Cook University, Townsville, QLD Australia; 8 Duke University Medical Center, Durham, NC USA; 9 Durham Veterans Affairs Medical Center, Durham, NC USA; 10 Department of Environmental Health Sciences Robert Stempel College of Public Health and Social Work, Florida International University, Miami, FL USA; 11 Department of Psychology & Neuroscience, Duke University, Durham, NC USA; 12 Department of Biological Sciences, Center for Human Health and the Environment, North Carolina State University, Raleigh, NC USA; 13 Department of Psychiatry and Carolina Institute for Developmental Disabilities, University of North Carolina at Chapel Hill, Chapel Hill, NC USA; 14 Department of Psychiatry and Behavioral Sciences, Emory University School of Medicine, Atlanta, GA USA; 15 Department of Epidemiology, Mailman School of Public Health, Columbia University, New York, NY USA; 16 Department of Psychiatry and Behavioral Sciences, Duke University, Durham, NC USA; 17 Departments of Psychiatry, Neuroscience, Molecular and Comparative Pathobiology, Johns Hopkins University School of Medicine, Baltimore, MD USA; 18 Developmental Mechanisms and Trajectories of Psychopathology Branch, National Institute of Mental Health, NIH, Rockville, MD USA; 19 Department of Epidemiology and Wendy Klag Center for Autism and Developmental Disabilities, Johns Hopkins University, Baltimore, MD USA

**Keywords:** Diseases of the nervous system, Genetic interaction

## Abstract

The etiologic pathways leading to neuropsychiatric diseases remain poorly defined. As genomic technologies have advanced over the past several decades, considerable progress has been made linking neuropsychiatric disorders to genetic underpinnings. Interest and consideration of nongenetic risk factors (e.g., lead exposure and schizophrenia) have, in contrast, lagged behind heritable frameworks of explanation. Thus, the association of neuropsychiatric illness to environmental chemical exposure, and their potential interactions with genetic susceptibility, are largely unexplored. In this review, we describe emerging approaches for considering the impact of chemical risk factors acting alone and in concert with genetic risk, and point to the potential role of epigenetics in mediating exposure effects on transcription of genes implicated in mental disorders. We highlight recent examples of research in nongenetic risk factors in psychiatric disorders that point to potential shared biological mechanisms—synaptic dysfunction, immune alterations, and gut–brain interactions. We outline new tools and resources that can be harnessed for the study of environmental factors in psychiatric disorders. These tools, combined with emerging experimental evidence, suggest that there is a need to broadly incorporate environmental exposures in psychiatric research, with the ultimate goal of identifying modifiable risk factors and informing new treatment strategies for neuropsychiatric disease.

## Introduction

Neuropsychiatric conditions such as anxiety, major depression, bipolar disorder, schizophrenia, obsessive-compulsive disorder, autism spectrum disorder (ASD), attention-deficit/hyperactivity disorder (ADHD), and post-traumatic stress disorder affect hundreds of millions of people worldwide. The economic and social costs are substantial [1, 2]. While much progress has been made in identifying the complex genetic contributions to these disorders [3], genetics alone does not fully explain risk and there is significant variation that can be ascribed to environmental factors. In the broadest sense, environmental exposures can be viewed as nongenetic factors that include psychosocial factors, diet and nutrition, pharmaceuticals and medical interventions, health conditions, infectious agents, environmental chemicals, and physical features of the environment. The contribution of some of these broadly defined factors, such as drugs of abuse and developmental risk factors (e.g., preterm birth and early-life adversity), to the pathogenesis of neuropsychiatric abnormalities have been well-studied (and discussed elsewhere) [4–7] compared to non-pharmaceutical chemicals that individuals are exposed to such as pesticides, metals, and air pollutants. In some cases, work in model systems has facilitated exploration of how environmental perturbations impact brain processes relevant to mental health. Such studies have furthered our understanding of vulnerability to neuropsychiatric disorders and revealed factors contributing to resiliency. Detailed investigation of the mechanisms and comorbidities associated with the development of neuropsychiatric disorders after exposure to environmental contaminants offers the potential to afford similar gains.

Emerging evidence has suggested that exposures to environmental chemicals can contribute to the pathogenesis of neuropsychiatric abnormalities. One of the few historical examples comes from occupational exposure to mercurous nitrate in the process for curing felt for the production of hats [8]. Prolonged mercury exposure led to “Mad Hatter” syndrome with symptoms including depression, delirium, and tremor [9, 10]. Other evidence comes from studies of lead (Pb), a prevalent exposure risk that has been known for many decades to cause cognitive impairments, even at low levels [11]. Indeed, for children, the US Centers for Disease Control and Prevention (CDC) currently maintains that “no safe blood lead level…has been identified” (https://www.cdc.gov/nceh/lead/default.htm). Pb exposures in childhood have been associated with specific neuropsychiatric disturbances, including symptoms of ADHD, anxiety, and depression [12–14]. A 17-year prospective birth cohort study in Chicago reported that each 1 µg/dL increase in blood Pb was associated with a 0.09 point increase in anxiety/depression symptom scores [15]. Deficits in executive function, a hallmark of many neuropsychiatric disorders, have also been linked to developmental Pb exposure in experimental systems [16]. Other studies have shown that developmental exposure to pesticides is associated with neuropsychiatric behavioral phenotypes [17, 18].

A deeper understanding of the risk of psychiatric disorders related to environmental exposures and the underlying biological mechanisms could lead to more effective prevention and treatment. This review summarizes how genetic and environmental factors, along with epigenetic modifications, are being investigated to assess susceptibility and progression of neuropsychiatric disease. Next, three exemplar biological targets of environmental exposures that may contribute to etiology of mental health disorders—synaptic integrity and transmission, immune function, and signaling along the gut microbiome-brain axis are described. Finally, key challenges that remain to be addressed to enable a rigorous interrogation of the potential importance of environmental agents as determinants of risk for the development of this diverse and heterogeneous constellation of disorders are outlined.

## The importance of including genetics in studies that assess environmental risks for neuropsychiatric disorders

Most psychiatric illnesses are considered to be complex diseases that involve genetic and environmental risk factors. Large genome-wide association studies (GWAS), made possible through the Psychiatric Genomics Consortium (https://www.med.unc.edu/pgc/about-us), have identified common genetic risk variants associated with specific psychiatric disorders [19–23], as well as across psychiatric disorders [24]. Common genetic risk variants for psychiatric outcomes are highly polygenic with many different genetic variants each contributing a small risk effect [25, 26]. Complementary whole-exome and genome-based studies have also identified a number of rare and/or de novo genetic variants that have larger risk effects on psychiatric outcomes. It is clear that genetic variation exerts a powerful influence on risk for major neuropsychiatric disorders; however, evidence as to the strength of these effects is conflicting. The wide variation in heritability estimates across and within neuropsychiatric disorders [27–29], and relatively small proportion of phenotypic variance explained by the genetic variants discovered to date, leaves substantial room for contributions from environmental factors in understanding neuropsychiatric risk.

The growing recognition that both genes and environmental factors contribute to neuropsychiatric disease risk underscores the need for studies that examine environmental exposures in the context of genetic risks, e.g., via gene–environment interactions (GxE). There are several conceptual models outlining how genes and environment can interact to influence disease susceptibility [30–34]; most neuropsychiatric studies have focused on the diathesis–stress interaction model [35], which postulates that inter-individual differences in genes result in different susceptibilities to environmental risk factors and candidate genes. Substance use disorders (SUDs) have been particularly proliferative in investigating GxE with numerous reports of interactions between social adversity or stress exposure and genetic variants that influence risk of drug or alcohol abuse [36–41]. Chemical exposures-gene interactions that influence risk of psychiatric and neurologic diseases have also been reported. For example, individuals with increased air pollutant or heavy metal exposure and a particular genetic variant have increased risk of neurodevelopmental disorders compared to individuals without the genetic variant, suggesting the genetic variant makes them more susceptible to the chemical exposure [42–46]. Interaction between cigarette smoking and genotype at the *PON1* gene locus were observed to influence the risk of bipolar and major depression disorders [47]. Studies that assess chemical exposures and a wider range of gene-environment conceptual models are needed to advance our understanding of gene–environment interactions in neuropsychiatric disease and better inform prevention, intervention, and treatment efforts.

Integration of genetic and chemical exposure data offers new opportunities in psychiatric research but there are numerous challenges that need to be recognized and properly addressed in GxE studies, including the need for large sample sizes, consideration of exposure complexities and measurement error, potential correlation between genetic and environmental factors, and use of appropriate study designs and statistical methods [48]. Researchers are beginning to address and overcome several of these challenges. First, studies based on unified genome-wide genetic and exposure information in large samples are emerging to facilitate the study of GxE in neuropsychiatric disorder pathogenesis. One such study is the Initiative for Integrative Psychiatric Research (iPSYCH), a consortium integrating data from national registries and genomic analysis of neonatal blood spots [49]. Many other large-scale studies, including work related to efforts of the Nordic OCD & Related Disorders Consortium (NORDiC), have been launched that will link genomic data with nongenetic exposures as contributors to mental health [50, 51]. In addition to launching new studies that collect both genetic and environmental information, experimental designs that can leverage substantial extant genomics data and biorepository resources would enhance GxE efforts. This will likely involve development of new cost-effective methods to enable rigorous ascertainment of environmental exposure data from individuals with extant genetic data. Statistical challenges in GxE include low power for traditional statistical methods and inconsistent results that have often failed to replicate in independent samples for both candidate gene and genome-wide GxE studies. Over the decade new statistical methods have been developed to improve power, appropriately control for type I error rates, address exposure misclassification and gene–environment correlation, and to ensure efficient implementation of GxE analyses [52, 53]. In addition, the recent development of polygenic risk scores, representing an individual’s genome-wide genetic risk as a single summed variable, provides opportunities to evaluate environmental exposure risks in the context of aggregate genetic risk using a well-powered approach. Finally, there are several additional complexities related to potential confounding and correlations between genetic and environmental exposures in human observational studies. Exposure mixtures and multiple forms of genetic risks (e.g., inherited common single nucleotide polymorphisms, rare de novo variants, DNA structural variants), need to be considered and potentially at different developmental or life course stages. In addition, genetic variation can result in different exposure to environmental risks. For example, single nucleotide polymorphisms (SNPs) located within the nicotinic receptor genes on chromosome 15 influence smoking behaviors [54]. Importantly, correlations between polygenic risk liability and environmental risk factors for psychiatric health outcomes have been reported [55, 56]. Thus, assessing gene-exposure correlations using unified genetic and environmental exposure data could provide important insights into the contribution of each risk factor, and their potential interplay, on psychiatric health outcomes. Complementary GxE studies in animal and cellular models provide an opportunity to overcome issues of confounding, timing, gene-exposure correlation, and exposure mixtures that are present in human observational data because they use highly controlled experimental conditions. Additionally, they can provide important mechanistic insights not always possible in human observational research.

## Epigenetics

Multiple lines of evidence support a role for epigenetics in the etiology of psychiatric disorders, particularly for chromatin remodeling and DNA methylation. Rare variant genetic studies of autism spectrum and bipolar disorders have shown chromatin remodeling genes are more frequently dysregulated among affected individuals [57–60]. Integration of genetic and epigenetic data has shown that genetic risk variants for ASD, schizophrenia, ADHD, and bipolar disorder control DNA methylation levels more often than non-psychiatric genetic variants [61–68]. Epigenetic changes have also been directly observed in postmortem brain [69–71], blood [72–74], semen [75, 76], saliva [77–79], placenta [80], and buccal [81–83] tissues in individuals with a psychiatric disorder. While these results support epigenetic involvement in psychiatric disease, there is some debate about the validity and reliability of these findings with respect to etiologic relevance. The concerns mainly stem from the inaccessibility of mechanistically relevant tissues or sorted cell types for brain disorders and the timing of sample collection to ascertain whether epigenetic changes are likely causal or consequential in the disease process. Because of the practical difficulty with addressing some of these concerns in human populations, complementary approaches using in vitro and animal-based model research will be necessary.

Epigenetic changes have also been associated with a diverse set of environmental exposures in human observational and animal model studies, including for chemical toxicants; these have been comprehensively reviewed elsewhere [84–88]. One of the best-known examples of a chemical exposure that directly alters epigenetic patterns comes from experiments in the Agouti mouse model. Exposure to bisphenol-A (BPA) in these mice leads to shifts in DNA methylation levels at the Agouti coat color locus and ultimately, an altered coat color [89]. BPA has also been associated with global and site-specific changes in DNA methylation and histone tail modifications in human observational studies [90–92]. A cross-organism study found mouse liver DNA methylation levels at the *STAT3* locus were altered in response to perinatal exposure to BPA and were also relevant to adult health outcomes [93]. The authors also found similar DNA methylation changes in *STAT3* related to BPA exposure levels measured in human fetal liver samples. Epigenetic changes in response to BPA, a type of endocrine disrupting chemical (EDC), are particularly relevant as an example given reports that EDC exposure may increase risk of psychiatric disease [94–96].

Studies of epigenetic changes related to chemical exposures and psychiatric disease have mainly been performed in parallel. Few studies have evaluated whether epigenetic changes related to environmental exposures are also associated with psychiatric disease or vice versa. Our ability to identify epigenetic changes related to both environmental chemicals and psychiatric disease and to formally evaluate potential epigenetic mechanisms in environmental effects on mental health is limited by small sample sizes, inaccessibility of brain tissue, potential exposure mixtures and confounding, and a lack of large prospective studies with unified epigenetic and environmental measures from relevant risk windows. This will require new epidemiology and animal and cellular model-system-based studies specifically designed to overcome these limitations. The National Institute of Environmental Health Sciences (NIEHS) is directly addressing several of these challenges through the Toxicant Exposures and Responses by Genomic and Epigenomic Regulators of Transcription (TaRGET) II Program [97]. Even if epigenetic changes are not in the causal pathway, they could provide a useful biomarker of current and past environmental exposures, including prenatal and early-life windows [98], or of psychiatric disease, which would also have great utility.

Beyond genetic risks, gene–environment interactions and epigenetic factors in psychiatric disorders, great progress has been made in understanding neurobiological mechanisms. Three examples of how environmental exposures may impact neurobiological substrates contributing to psychiatric disorders are discussed, including impacts on synaptic biology in schizophrenia, contributions of altered immune and microglial function to neurodevelopment, and emerging research on the microbiome and mood disorders. In each of these examples, environmental exposures can interact in multiple ways to influence function that contributes to risk for psychiatric disorders.

## Modification of synaptic transmission by environmental exposure: evidence from studies on lead and schizophrenia

Perturbations in synaptic integrity, synaptic plasticity and neurotransmission are implicated in the pathophysiology of several psychiatric disorders. Mice that lack actin depolymerizing proteins (ADF) and n-cofilin, which are critical for synaptic function, showed increases in locomotion and impulsive behaviors, as well as impairments in working memory [99]. In addition, many neural circuits require a balance between excitatory and inhibitory neurotransmitters with new treatments for mood disorders targeting modulation of gamma-aminobutyric acid (GABA) and glutamate neurotransmission [100]. In this section, we will use schizophrenia as an example to show the relevance of studying synaptic mechanisms when considering potential targets of environmental toxicants.

Aside from the longstanding dopamine hypothesis of schizophrenia pathogenesis, altered glutamate and GABA neurotransmission have also been implicated [101]. Substantial evidence suggests that alterations in glutamatergic function can produce changes in dopaminergic and GABAergic systems that may underlie the positive and negative symptoms associated with the cognitive components of the disease [102–104]. A currently accepted pathophysiologic framework for schizophrenia relates to hypofunction of the N-methyl-d-aspartate subtype (NMDAR) of glutamatergic ionotropic receptor during critical periods of brain development. This relative deficit in NMDARs results in alterations of neurobiological processes essential for brain growth, wiring, synaptic plasticity, and cognitive function, all of which are affected in schizophrenia [105].

While a body of prior work has examined the association of cannabis and tobacco smoking with schizophrenia risk, emerging evidence points to a role for environmental Pb exposure. As reviewed by Guilarte et al. [101], early-life Pb exposure in animals recapitulates behavioral, neurobiological, and imaging changes relevant to schizophrenia, including loss of parvalbumin-positive GABAergic interneurons (PVGI) in the frontal cortex, hippocampus, and striatum [106–108], and hyperactivity in the subcortical dopaminergic system [109, 110]. A recent rodent study by Stansfield et al. [111] found that chronic exposure to environmentally relevant concentrations of Pb (average 22 µg/dL) during early brain development produced selective loss of the PVGI phenotypic markers parvalbumin and glutamic acid decarboxylase 67 (GAD67) in prefrontal cortex, hippocampus, and striatum. Pb exposure also enhanced locomotor activity in response to the psychostimulant cocaine and increased striatal dopamine metabolites and D2-dopamine receptor binding, findings indicative of a hyperactive striatal dopaminergic system, as seen in model systems and postmortem brain from individuals with schizophrenia.

Population-based studies providing the first association of early-life (prenatal) lead exposure and schizophrenia were described by Opler and colleagues [112, 113]. Using two different population-based cohorts, they found that prenatal lead exposure (maternal blood lead levels estimated to be >15 μg/dL) were associated with a significant increase in the risk of schizophrenia later in life. Following these landmark studies there have been more recent reports providing additional support to this association. For example, a recent proof-of-concept study using childhood-shed teeth and laser ablation inductively coupled plasma mass spectrometry (LA-ICP-MS) showed higher levels of lead in teeth from diagnosed schizophrenic subjects relative to controls [114]. Furthermore, they found a positive correlation between early-life lead exposure and psychotic experiences in adulthood and a negative correlation with adult IQ. One of the advantages of the analysis of teeth using LA-ICP-MS is the ability to retrospectively reconstruct environmental exposures from the second trimester of pregnancy and through the first years of life. Using this approach, they found that the negative correlation of lead exposure with IQ was most pronounced if the exposure to lead occurred during the prenatal period. Arinola and colleagues also found increased plasma lead concentrations in newly diagnosed schizophrenic subjects [115]. Another study using a multidecade longitudinal design of lead-exposed children showed that higher levels of blood lead were associated with greater psychopathology and difficult personality traits as adults [116]. Childhood lead intoxication has also been associated with a high level of incarceration, delinquent behavior, and substance abuse, which are comorbid factors in schizophrenia [13, 117–119]. Lastly, recent assessments on the burden of lead exposure as a risk factor for mental illness in countries such as India have estimated that lead exposure accounts for a large portion of the prevalence of mental disorders including schizophrenia [120].

## Neuroimmune responses and environmental chemicals

Immune perturbations have attracted special interest for their possible link to psychiatric disorders [121–125], consistent with emerging knowledge that cytokines, chemokines and other immune factors, as well as the cells that produce them and/or harbor cognate receptors for them within the brain, play important roles in fetal brain development, brain maturation and brain function in adulthood. Notably microglia, resident immune cells of the brain, are critical players in normal brain development. In turn, aberrant immune signaling early in life may contribute to the pathogenesis of schizophrenia, ASD, and other neuropsychiatric disorders. This is consistent with the idea that early-life environmental exposures might affect later risk for psychiatric disorders through effects on the immune system. Research using maternal immune activation model(s) provides some support for this general idea, as do studies of exposure to chronic stress [126, 127]. Many chemical exposures are also known to interact with immune pathways and molecules, or contribute to the development of abnormal immune responses such as brain-directed autoimmune reactions, yet the linkage to alterations in brain development and the implications for psychiatric conditions remain largely unexplored [128–131]. This section summarizes recent work that points to microglia as an attractive target for further studies to advance this line of inquiry.

Microglial development begins with colonization of the brain by fetal yolk sac-derived macrophage precursor cells in early embryonic stages, followed by migration and spread throughout the central nervous system (CNS) [132, 133]. Microglia are critical for normal brain development via the phagocytosis of extraneous synapses [134–137] and apoptotic cells [138–140], learning-dependent synapse formation [141, 142], cortical wiring [143], neuronal survival [144], and the induction of programmed cell death [139, 145]. Owing to the critical role that microglia play in normal brain development, the activation or “programming” of these dynamic, long-living cells in response to immune activation, especially early in life, can have persistent consequences for brain function throughout the lifespan [146]. Maternal immune activation (MIA) in mouse dams in response to a viral mimic, Polyinosinic:polycytidylic acid (Poly IC), at GD 15 decreased microglial phagocytic function and altered behavior consistent with schizophrenia-like traits in humans, changes which were reversed using a partial microglial inhibitor, minocycline [147]. These data point to a potential critical role for microglia in persistent changes in neural function, although other studies have failed to show an impact on microglial function in MIA models [148]. This said, MIA models have typically been limited to infectious stimuli, whereas MIA is likely elicited by a broader range of environmental chemicals or stimuli, and we point to this possibility briefly in the sections that follow.

Psychosocial stress is a well-studied nongenetic risk factor for psychiatric disorders. Multiple stress models are associated with features of microglial activation, highlighting the role of these cells as key sensors of stress-related signaling by neurons [149]. Exposure to stress induces a chronically activated phenotypic profile within microglia—referred to as “priming”—during which time these cells undergo morphological changes, demonstrate pro-inflammatory bias, and exhibit an exaggerated response to experimental immune stimulation by endotoxins [150]. This primed or “sensitized” profile of microglia in psychological stress is implicated in the long-term re-establishment of behavioral symptoms such as anxiety-like behaviors in mice initially exposed to repeated social defeat stress [151].

There is now a significant body of literature linking air pollutant exposures with microglial activation and other neuroimmune changes, e.g., increased oxidative stress and lipid peroxidation [152–154]. The association between ambient air pollution and neurodevelopmental disorders such as ASD points to the prenatal period as particularly important. Indeed, recent studies of air pollution exposure underscore the ability of environmental chemicals to impact brain development via effects on microglia [155]. For example, maternal diesel exhaust particle (DEP) exposure exerted sex-dependent effects on fetal brain microglial morphology and development. Results in males were consistent with activation and/or a delay in maturation in several brain regions, along with striking changes in neural–glial interactions suggestive of a defect in phagocytosis [156]. In addition, exposure of pregnant dams to ultrafine particles (UFP), another component of air pollution, leads to increases in corpus callosum size, hypermyelination and microglial activation in offspring [157].

## Environmental exposures and the microbiome-gut-brain axis

The gut microbiome is increasingly understood to be a vital contributing component of the gut-brain axis. Mechanisms by which the microbiome may drive mental and brain health outcomes, and the need to understand how environmental chemicals can perturb these processes, are explored in this section.

Microbes and microbial metabolites influence neurobehavioral and neurotoxic outcomes through dysregulation of biological signaling pathways, or disruption of epithelial barrier integrity (“leaky gut”). Essential neurotransmitters for CNS functioning and mood regulation are interdependent with functions of the gut microbiome; for example, certain gut microbes are required to support peripheral production of serotonin and GABA, with serotonin serving as the key signaling molecule in both the enteric and central nervous systems [158, 159]. Critically, gut microbiota and metabolites, including short chain fatty acids, exert potent effects on peripheral immune processes via their effects on intestinal epithelial cells and modulation of G-protein coupled receptor-dependent signaling pathways [160]. In a leaky gut state, intestinal microbe membrane-derived lipopolysaccharide (LPS), also known as endotoxin, enters the peripheral circulation to promote an inflammatory state. Persistent low-grade immune-inflammatory processes have been implicated in the pathophysiology of major depressive disorder (MDD), schizophrenia, PTSD and mania [121, 122, 161–163], potentially linking the gut microbiome to psychiatric outcomes.

The role of environmental chemical exposure in gastrointestinal microbe–brain interactions is not well-characterized; however, inferential evidence in the literature supports microbial dysbiosis as a mediator of chemical-induced neurotoxicity. Toxicants may factor into the aforementioned mechanisms by targeting specific subpopulations of microbes to alter bacterial community diversity, creating or enhancing a leaky gut or neuroimmune state, or by undergoing biotransformation by bacteria to neurotoxic metabolites. The potential role of the gut-brain axis in chemical-induced neurotoxicity has been broadly reviewed previously [164, 165]. Despite the large body of literature pointing to interconnectivity of the microbiome, chemical exposure, and the brain, few studies have successfully linked all three factors in a manner that confers causality. This may be due to the many challenges faced by ascribing mechanism to something as variable as the microbiome.

Studies suggest that changes in intestinal microbiota diversity and community may be associated with psychiatric disorders such as ASD, schizophrenia, and depression [166–168]. While these studies demonstrate a clear gut–brain interaction, they do not consider the additional complexity of external environmental factors. A wide range of environmental factors and neurological outcomes have been linked to altered microbial composition. The anti-seizure benefits of a ketogenic diet were found to be dependent upon the microbiome in mice [169]. Light-stress in mice led to enhanced abundances of some bacterial species and metabolites, which correlated with reduced memory potential [170]. Some studies have focused on chemical stressors as a cause of microbial dysbiosis, although they tend to be more correlative in nature; for example, neurobehavioral changes due to developmental exposure to genistein, a phytoestrogen, were correlated with microbiome and metabolome changes in California mice [171].

The most significant challenge faced in understanding the role of the microbiome in psychiatric disorders, and human health more broadly, is moving beyond association and correlation to causation. This is not only true in rodents, but also in humans, where it is less evident, which factor is the mediator for disease and toxicity. Epidemiology studies have identified associations between microbial dysbiosis and environmental exposure, including traffic-related air pollution, persistent organic pollutants, mercury, lead and other chemicals [165, 172–175]. It is necessary to use a myriad of scientific approaches to arrive at a compelling display of evidence for gut microbiome–brain interaction. There have been several studies in rodents and humans examining this interplay in ASD. In an animal model of ASD, gut permeability was linked to serum levels of the microbial metabolite 4EPS; direct treatment of 4EPS was sufficient to induce autism-like behaviors in naïve mice [176]. Other studies have confirmed that autistic behaviors in mouse models of ASD can be attenuated by treatment with microbial metabolites or probiotics [177, 178]. A fecal microbial transfer (FMT) study in germ-free mice showed that mice harboring microbiota from human ASD donors, but not typical donors, displayed hallmark autistic behaviors [177]. In humans, there have been a few small studies assessing the utility of the gut microbiome to alter behavior. FMT in children with ASD who also presented with moderate gastrointestinal distress led to gastrointestinal and behavioral improvements [179]. Much more information is needed to understand which patient populations are most responsive, and whether probiotic treatment would be a valuable alternative. Importantly, there have also been many environmental exposures associated with the development of ASD [180–183], and more research into the role of the microbiome could help elucidate mechanisms.

ASD is just one example of a psychiatric outcome that may sit at the crossroads of microbial dysbiosis and environmental exposure. Continuing to clarify the relationship among environmental exposure, microbiome, and psychiatric outcomes is crucial because of the potential impact on human health and preventative medicine. Understanding these nuances can pave the way for developing novel interventions and biomarkers for exposure, gaining insight into inter-individual susceptibility, and ultimately allowing for focused, innovative, and holistic approaches to medical treatment.

## Addressing key challenges

The previous sections highlighted three areas, synaptic biology, immunology, and gut microbiome, where provocative new data support the feasibility and significance of studies to identify the role of environmental chemicals in psychiatric conditions. The pace of continued progress will depend on the ability of researchers to harness new tools and concepts that are being developed to understand causation of complex disorders. Foremost is the development and implementation of the exposome concept. Another important advance is the changing conceptualization of psychiatric disorders; the field is moving from symptom-based diagnosis to identifying patterns of neurobiologic dysfunction and dimensional models of psychiatric traits. New population-based preclinical models of common genetic diversity and computational resources such as the Comparative Toxicogenomics Database provide additional opportunities to explore relationships among chemicals, genes, and psychiatric disorders. This section lays out the promise of these new tools and approaches for the study of environmental contributors to psychiatric disorders.

Environmental health science is moving away from a focus on single toxicants or single classes of exposure, recognizing that we are exposed to complex mixtures that vary over time in composition and intensity. The concept of the exposome, proposed in 2005 as a complement to the genome [184], has evolved to be defined as the “cumulative measure of environmental influences and biologic responses throughout the lifespan” [185]. Three broad and partially overlapping domains of the exposome are recognized [186]. The specific external exposome includes exposures such as environmental chemicals, pharmaceuticals, radiation, infectious agents, diet and physical activity. The general external exposome refers to larger social and economic factors such as urbanicity, education, and climate. The internal exposome comprises the chemical milieu within the body and includes biologically active compounds associated with endogenous processes (e.g., inflammation, metabolism, microbiome). Significant advances in use and integration of data obtained from geographic information systems, remote sensing, wearable personal sensors, and numerous mobile phone-based applications (e.g., activity tracking) have created new opportunities for measuring important aspects of the external exposome [187]. Metabolomic-based biomonitoring has seen increasing use in measuring the internal exposome, as it can provide an untargeted, agnostic approach to characterize the holistic cellular response to exposure(s) in the context of susceptibility factors (e.g., aging, genetic variation). In the same way that genomic approaches provide significant advantage over candidate gene approaches to understanding the complex genetic architecture of psychiatric disorders, exposomic approaches can deepen our knowledge of the multiple, interactive and time-dependent nongenetic factors that affect risk and resilience for these disorders. While many measurement and analytic challenges remain, meaningful preparatory steps can be taken to ensure that psychiatric cohorts are poised to capitalize on advances in exposomics [188–190]. Moreover, collaborative efforts between psychiatric and environmental health researchers would provide an excellent testbed for addressing the integration of different aspects of the external exposome (e.g., environmental chemicals that are the focus of environmental health science studies and larger social and economic environments that are often considered in psychiatric research).

The current phenomenological criteria used to clinically diagnose an individual with mental illness is subjective [191], leading to considerable variance and imprecision [192, 193]. For research purposes, this means that individuals with distinct neurobiological circuits and pathophysiology may be grouped together under a single diagnosis, obscuring important differences in risk factors among individuals. The corollary, that two individuals with different clinical diagnoses may have shared pathophysiology, also creates challenges for clinical research. To help address this issue, the National Institute of Mental Health (NIMH) has spearheaded an initiative to classify neurocircuit mediated symptoms, with relevance to psychiatric disorders, by creating the Research Domain Criteria (RDoC) initiative [194, 195]. Although studies of environmental influences are strongly emphasized and encouraged in RDoC research (https://www.nimh.nih.gov/research/research-funded-by-nimh/rdoc/constructs/rdoc-matrix.shtml), specific environmental effects are not listed explicitly. New partnerships between psychiatric and environmental health researchers are needed to implement RDoC for the study of nongenetic/environmental influences. Psychiatric researchers who are already collecting data in the framework of RDoC domains and constructs can expand their studies to include one or more exposures, thus contributing data to illuminate whether and how relationships among functional domains and constructs may depend on environmental context. Environmental health researchers can consider how the RDoC framework can be incorporated in their own lines of research; this may require additional collection of physiology, behavior, cognition, and symptom measures to enable systematic mapping to existing RDoC domains and constructs. Ultimately, the use of objective measures anchored to neurocircuits of neuropsychopathology as in RDoC will allow for more precise evaluation of environmental contributions to psychiatric symptoms and disease.

The increasing recognition of psychiatric disorders as dimensional rather that dichotomous [196–199] also has implications for the study of nongenetic influences. The low prevalence of most psychiatric disorders requires very large sample sizes for sufficiently powered population-based studies. Unfortunately, deep and rigorous exposure assessments on large numbers of study participants are often not feasible; however, if psychiatric traits are distributed continuously in the general population, it is possible to use an unselected sample to identify associations of exposures with variation in the trait because, in addition to individuals that meet diagnostic criteria, the much larger number of individuals in the population-based sampling frame with subclinical traits can contribute to identifying any association with exposure. This approach could be adapted for use in many existing smaller studies that have deep exposure characterization. The Epidemiology Resources web tool (https://tools.niehs.nih.gov/cohorts/) organizes and shares information about environmental epidemiology studies supported by the NIEHS, with the goal of facilitating new collaborations and ancillary studies such as those recommended in this section.

Model systems provide a critical tool in the investigation of environmental exposure effects on human health. Although rodents cannot fully recapitulate the complex neuropathology of psychiatric disorders, model systems can be used to test hypotheses about the impact of perturbations representing potential risk factors, including genomic or environmental variables, on brain processes relevant to mental health. Thus, these models can provide a platform to investigate the role of environmental mechanisms that shape neurodevelopment of circuits linked to etiology and symptomatology of human psychopathology. Although models that focus on a single exposure and/or genetic risk are prevalent, a promising trend is the use of models that better reflect multifactorial risk. “Two-hit” rodent models have been used to understand the interactions between multiple or repeated exposures at critical developmental timepoints. For example, a second juvenile exposure to polychlorinated biphenyls (PCBs) can have profound and differential sex-specific effects on gene expression, social or anxiety behavior in rats prenatally exposed to the same PCBs [200, 201]. Many other “two-hit” models, often focused on immune and/or stress exposures, support the important role of combined exposures in psychiatric phenotypes [202–204].

As was described in an earlier section, both rare and common forms of genetic risk contribute to psychiatric disorders. A recent GWAS study identified more than 100 genetic loci for schizophrenia [21]. The large number of common alleles confer modest effect; this suggests that reliance on models with disruption of single genes to test gene–environment interaction may not be informative. To confront the challenge of polygenic causation of complex diseases, population-based mouse models have been developed [205]. The Collaborative Cross (CC) is a large panel of recombinant inbred mouse strains intentionally designed to mimic the genetic diversity observed in human populations [206, 207]; this provides a powerful model to support systems genetic approaches for the study of quantitative biologic and behavioral traits. In the context of environmental exposures, studies using population-based mouse resources such as CC have the advantage of including both mice with increased sensitivity and those with reduced sensitivity (resilience). CC mice can be used to study gene–environment interaction, i.e., how common genetic variation modifies the effect of exposure(s) on psychiatric phenotypes. As these mice capture a very wide range of common genetic variation, researchers are freed from focusing on a preselected small number of candidate genes/loci thought to confer susceptibility, making it more likely that new, previously unrecognized GxE will be identified. Two recent examples in psychiatric research support the use of CC mice to study gene–environment interactions [208, 209].

As emphasized throughout this section, new approaches are needed to move beyond single gene and single exposure studies of mental illness. Data generation is increasing in areas of brain-based disorders, and spans genomics, transcriptomics, metagenomics, and exposomics. Given the diversity of information scope and scale, providing resources to mine, share and manage relevant information will require public resources and data standards. One resource that merits attention is the Comparative Toxicogenomics Database (CTD) [210]. CTD is a manually curated database that provides information describing chemical-gene interactions, chemical- and gene-disease relationships, chemical-phenotype relationships and detailed exposure data [211–214]. These data are integrated with pathway and functional information with tools that can help users inform hypotheses about the etiologies of phenotypes and diseases with a specific focus on environmental and exposure influences. In addition to curation and summation of data providing direct evidence for linkage of a gene or exposure with a disease, CTD provides statistically ranked inferred relationships among genes, exposures and diseases. This feature is particularly powerful for unearthing new clues about possible chemical-disease relationships. For example, if the curated literature indicates that an exposure influences the expression of a gene and, in separate publications, that gene is associated with a disease, then CTD provides an inference (via the shared gene) that the exposure is linked to the disease; this provides a new testable hypothesis that can be pursued further in laboratory or clinical settings. CTD has been employed in the analysis of a range of brain disorders [215–218] and can be helpful for guiding future hypotheses about relationships among exposures, genes and psychiatric disorders.

## Concluding perspective

The landscape of psychiatric research has changed rapidly over the past decade. The growing evidence that environmental exposures disrupt normal brain and behavioral function to increase psychiatric risk suggests that greater collaboration between psychiatric and environmental health research communities is critical. Further, strategies to determine the most appropriate and productive directions in psychiatric research will undoubtedly require the inclusion of and appreciation for emerging areas discussed here, e.g., synaptic plasticity, immunology, gut microbiome-brain signaling, and others. The potential payoff for identifying environmental risk factors for psychiatric disorders is tremendous, as it affords a unique opportunity to not just modify symptom trajectory following diagnosis, but the possibility to prevent the development of the disease from the outset.

## Funding and disclosure

This work was supported in part by the NIH, National Institute of Environmental Health Sciences (to JA Hollander, KA McAllister, AL Dzierlenga, AE Garton, CP Lawler) and National Institute of Mental Health (to JL Zehr), the NIH grants: R01ES014065 and P30ES025128 (to CJ Mattingly); R01ES06189 (to TR Guilarte); R01HD090051 (to M Hornig); U54HD079124 (to SS Moy); and the U.S. Department of Veterans Affairs grant IK2CX001397 (to ST Szabo). FN Jacka has received Grant/Research support from the Brain and Behavior Research Institute, the National Health and Medical Research Council (NHMRC), Australian Rotary Health, the Geelong Medical Research Foundation, the Ian Potter Foundation, Eli Lilly, Meat and Livestock Australia, Woolworths Limited, Fernwood Gyms, The Wilson Foundation, The A2 Milk Company, The University of Melbourne and has received speakers honoraria from Sanofi-Synthelabo, Janssen Cilag, Servier, Pfizer, Health Ed, Network Nutrition, Angelini Farmaceutica, Eli Lilly and Metagenics. FN Jacka has written two books for commercial publication that promotes the idea that good diet quality is important for mental and brain health. The views expressed in this review are those of the authors and do not necessarily reflect the position or policy of the Department of Veterans Affairs or the United States Government. The remaining authors declare no competing interests.
